# *Dnd1* Knockout in Sturgeons By CRISPR/Cas9 Generates Germ Cell Free Host for Surrogate Production

**DOI:** 10.3390/ani9040174

**Published:** 2019-04-17

**Authors:** Abdul Rasheed Baloch, Roman Franěk, Tomáš Tichopád, Michaela Fučíková, Marek Rodina, Martin Pšenička

**Affiliations:** Faculty of Fisheries and Protection of Waters, South Bohemian Research Center of Aquaculture and Biodiversity of Hydrocenoses, University of South Bohemia in Ceske Budejovice, Zátiší 728/II, 389 25 Vodňany, Czech Republic; franek@frov.jcu.cz (R.F.); tichopad@frov.jcu.cz (T.T.); fucikova@frov.jcu.cz (M.F.); rodina@frov.jcu.cz (M.R.); psenicka@frov.jcu.cz (M.P.)

**Keywords:** Acipenser, caviar, conservation, genome editing, morpholino oligonucleotide, PGCs

## Abstract

**Simple Summary:**

Sturgeons, also called archaic giants, are critically endangered fish species due to overfishing for caviar and interference in their natural habitats. Some sturgeon species have life spans of over 100 years and sexual maturity is attained between 20 to 25 years. Sterlet (*Acipenser ruthenus*) has fastest reproductive cycle; thus, this species can be used for surrogate production in sturgeons. Primordial germ cells are the origin of all germ cells in developing embryos. *Dnd1* is essential for formation and migration of primordial germ cells and its inactivation results in sterility in fish. In our study, we have used a cutting-edge genome editing technology known as CRISPR/Cas9 to knockout *dnd1* and to prepare a sterile sterlet host. CRISPR/Cas9 knocked-out embryos lacked primordial germ cells and can be used as a sterile host for surrogate production in sturgeons.

**Abstract:**

Sturgeons also known as living fossils are facing threats to their survival due to overfishing and interference in natural habitats. Sterlet (*Acipenser ruthenus*) due to its rapid reproductive cycle and small body size can be used as a sterile host for surrogate production for late maturing and large sturgeon species. Dead end protein (dnd1) is essential for migration of Primordial Germ Cells (PGCs), the origin of all germ cells in developing embryos. Knockout or knockdown of *dnd1* can be done in order to mismigrate PGCs. Previously we have used MO and UV for the aforementioned purpose, and in our present study we have used CRISPR/Cas9 technology to knockout *dnd1*. No or a smaller number of PGCs were detected in crispants, and we also observed malformations in some CRISPR/Cas9 injected embryos. Furthermore, we compared three established methods to achieve sterility in sterlet, and we found higher embryo survival and hatching rates in CRISPR/Cas9, UV and MO, respectively.

## 1. Introduction

Sturgeons are an ancient fish species that have existed for at least 200 million years and are famous for their caviar [1]. Their value as a source of caviar has led sturgeons to be target of intensive legal and illegal fisheries, therefore resulting in the collapse of several sturgeon species and stocks [2,3]. Natural populations of these archaic giants have been declining due to certain factors such as water pollution and interference in their natural habitats. Other prominent impacts on the sturgeon population are hybridization, introduced species, water divergence, reduced food supply and saltwater intrusion [4,5]. Damming of rivers has also been proved to be detrimental to sturgeon populations because it reduces and/or eliminates spawning and egg/larvae habitats [6]. According to the International Union for the Conservation of Nature (IUCN 2010), 85% of sturgeon species are at the verge of extinction. Sturgeons are generally a long living species and exhibit late onset of maturity, and a slow growth rate with infrequent reproduction [2]. Some sturgeon species have a life span over 100 years and sexual maturity is attained between 20 to 25 years or even later in females [3]. Sterlet (*Acipenser ruthenus*) has the fastest reproductive cycle; males mature over 3 to 7 years and females over 5 to 9 years [7], therefore making it convenient to produce donor-derived gametes of large and long maturing sturgeons. Surrogate production by the generation of germ line chimera through germ cell transplantation in various closely related species has been established in several fish species [8,9,10,11,12,13,14,15,16]. A germ cell free host is a pre-requisite for surrogate production, and host gonads lacking germ cells could improve transplantation efficiency, as the niche is not occupied by the endogenous germ cells and germ-line chimera only produce donor derived gametes. Triploidization is practically used in species whose triploid individuals are sterile [17,18,19]. Nevertheless, sturgeons are evolutionary polyploids and sterility of triploids is not yet well proved [20], therefore alternatively, other methods should be used to achieve sterility in sterlet.

Inactivation of mRNAs such as *dead end* (*dnd1*) that is essential for formation and migration of PGCs should be done to achieve sterility experimentally, as was already shown in zebrafish, medaka, loach, goldfish and sturgeons [21,22,23,24]. Dnd1 in vertebrates binds to 3’-UTRs (untranslated regions) of germ cells specific RNAs, thus it protects them against miRNA-mediated degradation so that these RNAs can contribute to maintain the fate of PGCs [25,26]. Loss of *dnd1* in mouse also results in germ cell free sterile gonads [27]; however, in mammals, zygotic transcription replaces maternal RNA at the 1 cell stage, while in fish it occurs later during mid-blastula transition [28]. This feature affects the ability of some maternal RNAs to maintain germ cells like *piwil1*, *piwil2* and *vasa* that start germ cells formation in homozygous mutant fish for aforementioned genes; however, the loss of germ cells occurs at later stages [29,30,31]. Certain studies in fish have assayed function of *dnd1* when both maternal and zygotic *dnd1* mRNA is lost during early development [21,22,32]. Zygotic gene expression is not turned on until the onset of gastrulation in fish; and zygotic *dnd1* RNA in salmon was found to be required for germ cells migration to gonads [33]. In tilapia, CRISPR/Cas9 KO of *nanos3* gene resulted in germ cell free gonads, therefore further strengthening the notion that maternally contributed factors cannot rescue germ cell development and survival [34]. Loss of germ cells in fish affects the somatic sex of gonad differently since PGCs loss leads to an all-male phenotype in medaka, zebrafish and tilapia, while both males and females develop in germ cell-free loach and goldfish [21,22,23,34,35]. In mice, *dnd1* knock out leads to all-male offspring. It is still unclear whether or not the absence of germ cells is relevant for the differentiation of sex in sturgeons.

The latest techniques of genome editing can insert, delete and/or alter DNA sequences of cells and organisms, thus enabling scientists to dissect functions of specific genes [36]. In CRISPR/Cas9, RNA guided endonucleases (Cas9) from microbial adaptive immune system CRISPR can be easily targeted to virtually any genomic location of choice by short RNA guide. This genome editing system presents advantages over other genome editing technologies (ZFNs and TALENs) such as high efficiency, convenience and cost-effectiveness [37,38]. The CRISPR/Cas9 system therefore, is suitable to be applied in new fish models to generate sterile host by targeting the germ-cell specific candidate gene approach [26]. Previously we have used morpholino oligonucleotide (MO) against *dnd1* in sterlet to achieve sterility [24] and depleted PGCs from sturgeons’ embryos by UV irradiation [39]; however, it needs to inject many embryos with MO, and dechorionation is required for UV irradiation, which is laborious. In our present study, we have knocked out *dnd1* in sterlet by applying CRISPR/Cas9 technology.

## 2. Materials and Methods

### 2.1. Ethics Statement

All animal experiments were conducted in accordance with the Animal Research Committee of Faculty of Fisheries and Protection of Waters in Vodňany, University of South Bohemia in České Budějovice, Czech Republic. All experimental fish were maintained according to principles based on the European Union (EU) Harmonized Animal Welfare Act of the Czech Republic, and Principles of Laboratory Animal Care and National Laws 246/1992 “Animal Welfare” on the protection of animals were followed. Experiments were approved by the Ministry of Agriculture of the Czech Republic (reference number: MSMT-12550/2016-3).

### 2.2. Fish Source, Preparation of Embryos and Collection of Samples

During the spawning season (March to June 2017), females and males of adult sterlet (*Acipenser ruthenus*) of five to nine years of age were transferred from outdoor ponds into recirculating aquaculture system installed indoors. Fish were kept in tanks of 4000-L and water temperature was increased to 15 °C. In order to induce spermiation, male sterlet were injected with single intra-muscular injection of carp pituitary extract at 4 mg/kg body weight (BW) in 0.9% NaCl. Sperm collection was done 48 h after hormonal injection and were kept at ice at 4 °C until fertilization. Light microscopy was used to assess motility of spermatozoa that was found to be more than 80% and were used for the fertilization. Carp pituitary extract was used to stimulate the ovulation by intra-muscular injection in two doses, i.e., first dose at 0.5 mg/kg BW and second at 4.5 mg/kg BW, 12 h after the first injection. Collection of ovulated eggs was done from three different females from 18 to 20 h after the second injection, and eggs were inseminated with sperm from two different males at 15 °C in dechlorinated water. To remove stickiness, eggs were three times rinsed in 0.04% tannic acid. One hour later after the fertilization, chorion membrane (outer layer of eggs) was removed by using forceps. Chorion removed eggs were then transferred into 100 mL dechlorinated tap water with 0.01% penicillin and streptomycin in the glass petri dishes. Embryos were incubated at 15 °C in incubator. Temperature regulation was done at 15 ± 1 °C throughout the experimental period and water was changed on daily basis. Embryos were used for injection of sgRNAs and Cas9 complex to knockout *dnd1*, isothiocyanate (FITC)-biotin-dextran (molecular weight = 500,000) in order to label PGCs [40], antisense MO for the depletion of PGCs [24], UV irradiation for PGCs removal [39] and polymerase chain reaction. Embryos were kept at −80 °C for the extraction of DNA for downstream applications.

### 2.3. Cas9 Protein and sgRNAs

Five single guide RNAs (sgRNAs) were designed to target *Acipenser ruthenus dnd1* (*Ardnd1*) gene. Target sites were, sgSRNA1: GGGGGGAATGCAGTCCAACC; sgRNA2: GGGGGAATGCAGTCCAACC; sgRNA3: TTCAATCATTTTCTTTCTTA; sgRNA4: TGGTTTAAAACCGTAAAGAT and sgRNA5: ATTTTCTGAGTCCATGTTTC. Oligos for sgRNAs were ordered from Macrogen Company (Macrogen Inc., Amsterdam, the Netherlands) and were annealed according to references [41,42] (Figure S1). In vitro transcription (IVT) using HiScribeTM T7 High Yield RNA synthesis kit (NEB) was used to generate sgRNAs, according to manufacturers’ instructions. Synthesized sgRNAs were treated with DNAse to remove any remaining DNA traces and mySPEC spectrophotometer (VWR^®^ mySPEC spectrophotometer) was used for sgRNAs quantification. All sgRNAs were diluted and aliquoted. Cas9 protein was purchased from PNA Bio and was re-suspended as per manufacturers’ instructions, aliquoted and stored at −80 °C.

### 2.4. Microinjection of sgRNA and Cas9 Complex

Approximately 50 fertilized eggs of sterlet were aligned in each petri dish; in total 600 embryos were injected. Embryos at the 1 cell stage were injected with prepared complex of mixture all five sgRNAs and Cas9 protein (gRNAs+Cas9 ribonucleoprotein complex) in animal pole using a glass capillary needle. 1% FITC-biotin-dextran (molecular weight = 500,000) was co-injected in vegetal pole to label PGCs, and in the control group only 1% FITC-biotic-dextran was injected according to Saito et al. [43]. Glass micropipette was drawn from a glass needle (Drummond, Tokyo, Japan) using a needle puller (PC-10; Narishige, Tokyo, Japan). Microinjection was done under fluorescent stereomicroscope Leica M165 FC (Leica, Wezlar, Germany) with a pressure of 100 hPa for ~1 second. After microinjection of sgRNAs+Cas9 ribonucleoprotein complex, the survival rate and number of FITC labeled PGCs were examined at four days post fertilization (dpf). At 22 dpf, larvae were taken from each group, euthanized by using tricaine solution overdosing, body cavity of larvae was opened, dissection of the gut was performed and the position of PGCs were observed and their number was counted precisely. Embryos were kept at 15 °C, hatched larvae were fed with Tubifex. Embryo development was recorded with steromicroscope Leica M165 FC with camera (Leica DFC425C). Embryos and larvae from each group were kept in −80 °C for downstream applications.

### 2.5. Preparation of Genomic DNA

Control sterlet embryos and sgRNA+Cas9 ribonucleoprotein injected embryos were individually collected after 4 dpf and 22 dpf of injection. Total genomic DNA was isolated using a PureLink Genomic DNA Mini Kit (Invitrogen). Primers (F: GAGAGGGCAAGTTGTCTGGA; and R: AAAACCTCACAGCCAGAGGAA) were used to amplify the region of *Ardnd1* gene spanning the target sites. Mutation detection assay was performed according to [44,45] and Multina-500 (Shimadzu) according to reference [46] was used to detect the mutations.

### 2.6. Capillary Electrophoresis and Mutation Detection Assay

In order to perform the mutation detection assay (HMA; heteroduplex mobility assay), PCR on the genomic DNA was run over 35 cycles using KOD FX Neo (TOYOBO) and PCR products were analyzed using a microchip electrophoresis system (DNA-500 reagent kit and MCE-202 MultiNA; Shimadzu) according to Shigeta et al., 2016 [47].

### 2.7. Statistical Analysis

Statistical significance of treatments on number of PGCs was analyzed by Wilcoxon test and Kruskal-Wallis test. If there was a significant difference, thereafter we used Dunn’s Post-Hoc test to find out which groups were different (*p* < 0.05). Statistical tests were performed using R programming language software (Version 3.5.1, 2018).

## 3. Results

### 3.1. sgRNA/Cas9 Ribonucleoprotein

**Figure 1:**
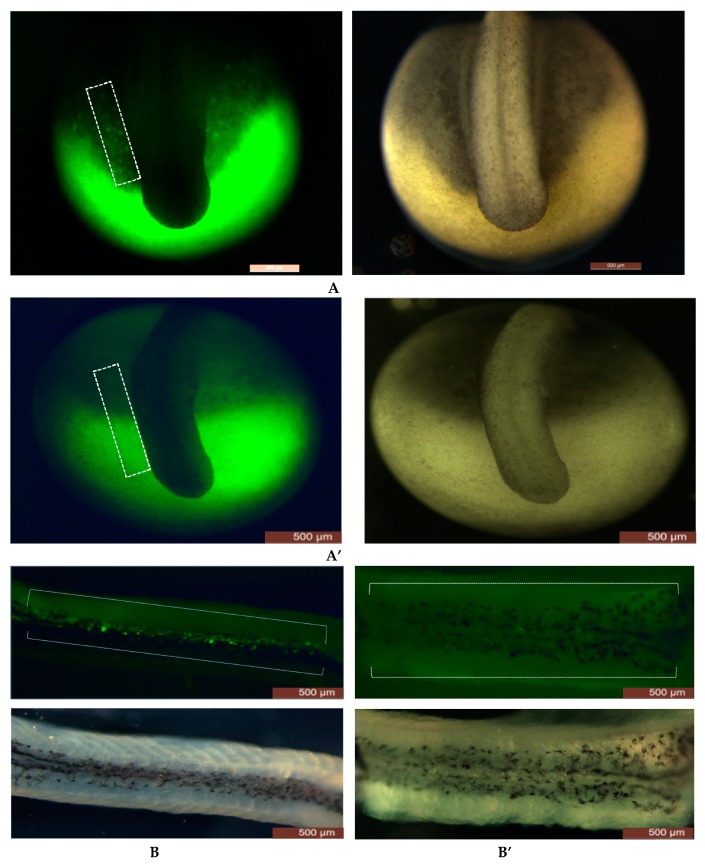

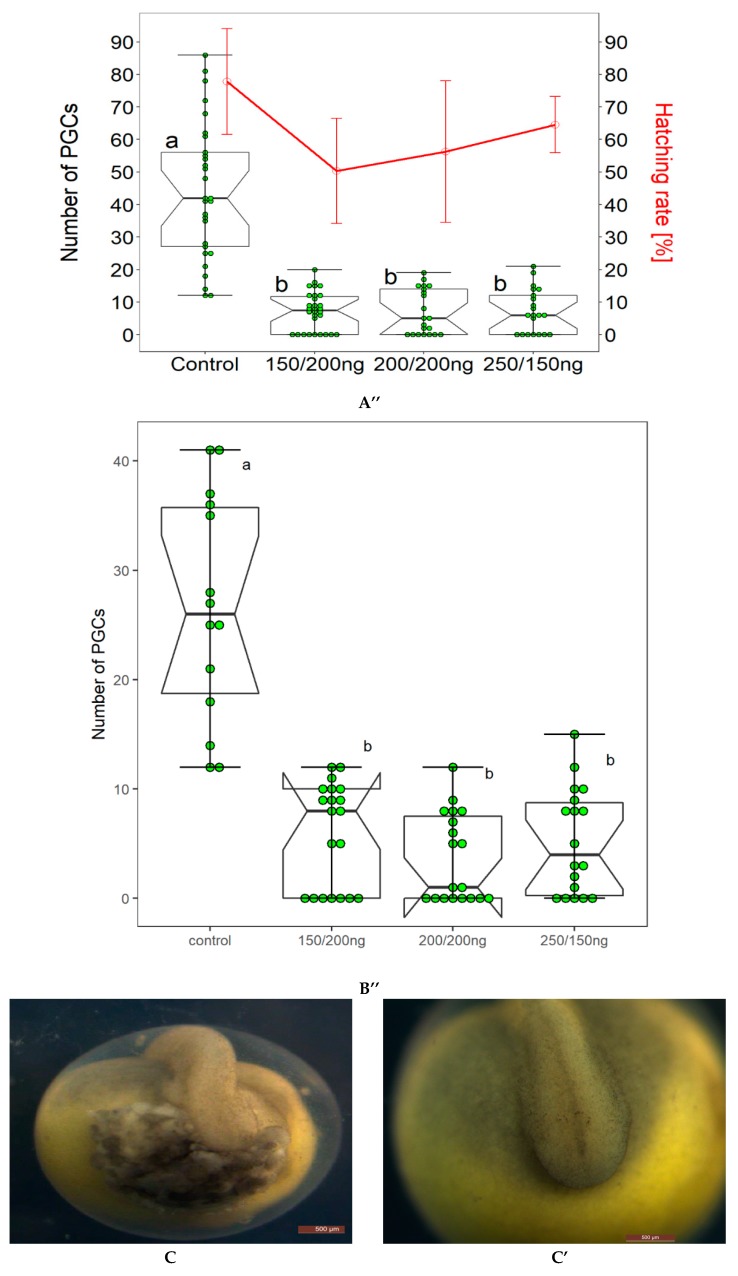
Number of FITC-labeled PGCs reduced from neurula stage to tail bud stage after embryos were injected with RNPs (sgRNAs+Cas9 protein). (**A**) Control group and RNPs injected group with FITC-dextran to label the PGCs at tail bud stage [40], respectively. Embryos injected with RNPs (**A’**) are lacking the FITC-labeled PGCs around surround the region where tail develops; however, in control group (**A**) FITC-labeled PGCs can be easily seen. (**A’’**): Different concentrations of sgRNAs and Cas9 protein were used to find optimal concentrations; however, no significant difference of sgRNAs/Cas9 concentrations was found on FITC-labeled PGCs (box graphs). Hatching rates were also found to be insignificant (line graph). (**B**,**B’**): In fluorescent image, many FITC-labeled PGCs can be seen in gonadal region (under a white broken line) in the control group (**B**); however on the contrary no FITC-labeled PGCs can be seen at the same region when injected with RNPs complex (B’; under white broken line). Number of FITC-labeled PGCs were observed by opening the body cavity of larvae, FITC-labeled PGCs were found to be in lower number when different concentrations of sgRNAs/Cas9 were used; while in the control group, FITC-labeled PGCs were found in a higher number (**B’’**). (**C**) Malformed embryo at 4 dpf after the injection of RNPs (in one batch of eggs from one female) and the non-injected control group (**C’**). The boxes with different letter show the significant difference (*p* < 0.05).

In our present study, no significant difference was observed in survival and hatching rates of embryos injected with different concentrations of gRNAs and the Cas9 protein (Figure 1A’’). PGCs in FITC labeled control group and gRNAs+Cas9 ribonucleoprotein complex injected group with different concentrations of sgRNAs and Cas9 protein were counted at 4 dpf and 22 dpf. At 4 dpf, almost no PGCs and/or a smaller number of PGCs were observed compared to the control group (Table 1, Figure 1A’). After 22 dpf, larvae from the gRNAs+Cas9 ribonucleoprotein complex injected group and control groups were euthanized and dissected and the number of PGCs was analyzed in body cavity. PGCs were found to be colonized where the genital ridge presumed to be localized (Figure 1B). gRNAs+Cas9 ribonucleoprotein complex injected group contained significantly lower number of PGCs than control group, and moreover, PGCs were not detected in dissected body cavities of crispants that were lacking PGCs at 4 dpf (Table 2, Figure 1B’). Intriguingly, eggs from one batch were found to be malformed such as having a cloudy structure at the region where the PGCs were supposed to have originated, when injected with sgRNA+Cas9 ribonucleoprotein (Figure 1C). These embryos did not hatch and ultimately died in chorion membrane.

### 3.2. Comparison of CRISPR/Cas9, Dnd1-MO and UV Irradiation

**Figure 2:**
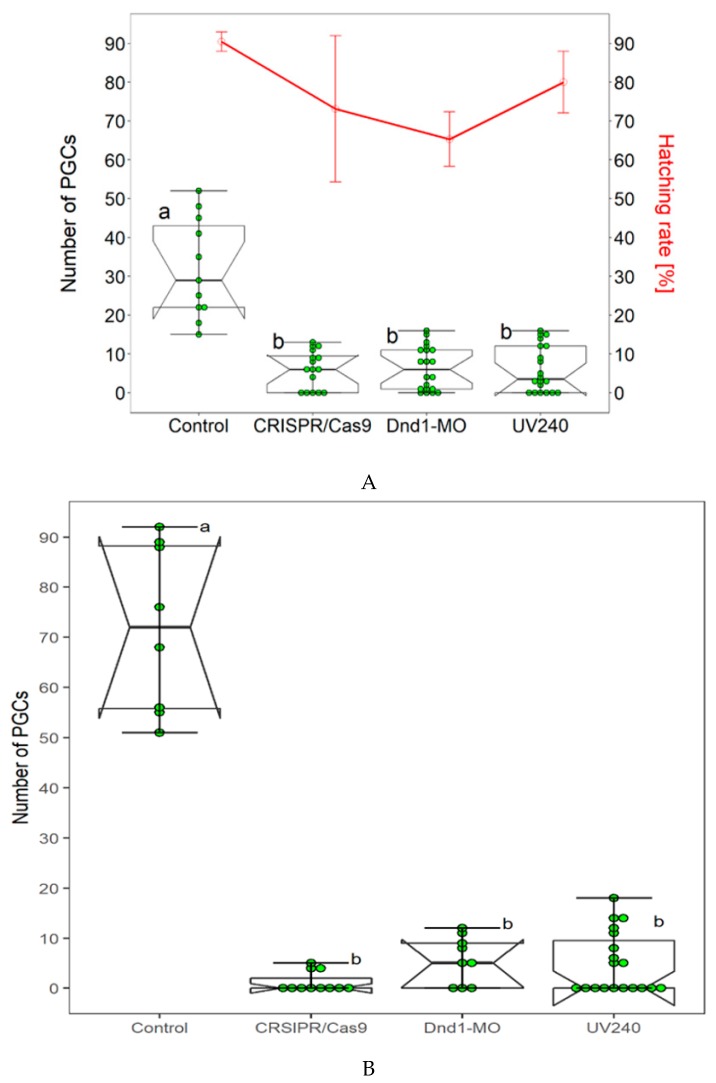
Three different methods to achieve sterility in sterlet (*Acipenser ruthenus*) have been established and were compared. (**A**): The control and embryos injected with CRISPR/Cas9, *Dnd1*-MO and UV240 exposed embryos; injected with FITC-dextran to label PGCs. Number of PGCs were counted in all three treated groups i.e., CRISPR/Cas9, *Dnd1*-MO and UV240, and in control group. FITC-labeled PGCs were found to be reduced at the neurula to tail bud stage (box plots). Hatching and survival rates were found to be lower in *Dnd1*-MO embryos (line graph) when compared to the control group. In CRISPR/Cas9 and UV240 irradiation method, hatching and survival rates of embryos did not significantly vary (line graph). The survived embryos from all three treated groups were kept and FITC-labeled PGCs were observed at 22 dpf. Number of FITC labeled PGCs continued to be absent in CRISPR/Cas9, *Dnd1*-MO and UV240 groups, while larvae from the control group have many FITC-labeled PGCs in the gonadal region (**B**). Bars with different letters signify a significant difference (*p* < 0.05).

We have already successfully used the *Dnd1*-*MO* technology and UV irradiation to achieve sterility in sterlet. In our present study, we compared two aforementioned methods with CRISPR/Cas9. *Dnd1*-*MO* technology was applied according to Linhartova et al., [24]. *Dnd1*-*MO* caused complete removal of PGCs from the injected embryos (Table 2, Figure 2A); however, average lower survival and hatching rates were found in embryos and larvae at 4 and 22 dpf (Figure 2A) as compared to the control. We then studied the effect of UV irradiation on number of FITC-labeled PGCs by exposing the embryos at 240mJ/cm^2^ (UV240) according to Saito et al., [39]. In group of embryos that were exposed to UV240, few ectopic FITC labeled PCCs were detected in some exposed embryos (Table 2, Figure 2A). However, most of exposed embryos were lacking the FITC-labeled PGCs in the surrounding region of the tail bud, where they were expected to accumulate. Embryos that were exposed to UV240 developed normally and grew up healthy until 22 dpf when they were euthanized to count number of PGCs in body cavity (Figure 2B). In the CRISPR/Cas9 experiment, number of PGCs was counted at 4dpf and 22 dpf. FITC-labeled PGCs were rarely found in genital ridges of CRISPR/Cas9 treated embryos. Compared to three aforementioned methods, number of FITC-labeled PGCs at 22 dpf in control group increased when compared those in neurula stage. No significant difference in hatching and survival rates of embryos were found in the three compared methods.

### 3.3. Capillary Electrophoresis and Mutation Detection Assay

**Figure 3:**
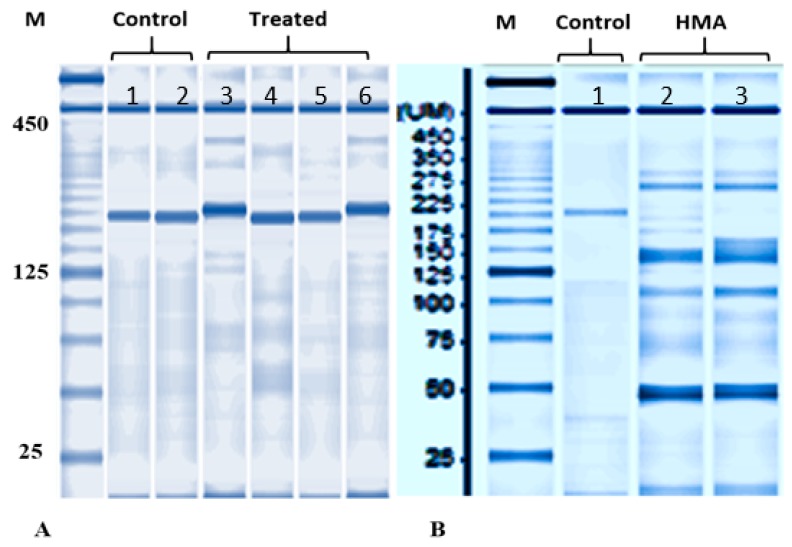
Genotyping by HMA assay in the un-injected and injected sterlet embryos. PCR products encompassing sgRNAs target sites were analyzed using a microchip electrophoresis system. (**A**) Bands from DNA of two injected embryos (sample number 3 and 6) were found to have different size; (**B**) HMA of two embryos having different bands (sample number 2 and 3). The sample 2 and 3 in Figure 3B were numbered as 3 and 6 in Figure 3A. Heteroduplex bands and multiple short bands are shown in sgRNA+Cas9 RNP injected embryos (crispants; B: sample 2 and 3).

Normally developed embryos lacking PGCs were collected to examine the efficiency of disruption at on-target sites at 4 dpf. By using capillary electrophoresis some samples were found to have different band sizes (Figure 3) when compared with non-injected embryos. In mutation detection assay, different bands formation was detected in aforementioned samples, indicating that disruption of target sites occurred for *Ardnd1* (Figure 3). As expected, several shorter bands were observed in embryos injected with mixture of sgRNA+Cas9 ribonucleoprotein. Injection of sgRNA+Cas9 ribonucleoprotein presumably induced frameshift mutations and/or small deletions in the *dnd1* gene [33,46,47,48].

## 4. Discussion

The majority of the genome editing studies have been conducted on polyploid species in plants such as rice (*Oryza sativa*), sugarcane (*Saccharum* spp. *hybrids*), *Camelina sativa* and *Arabidopsis thaliana* [49,50,51]. In fish model species, zebrafish, the CRISPR/Cas9 research with codon optimization of Cas9 mRNA showed induction of mutation with 75 to 99% rates in endogenous genes [52]. However, same method does not induce more than 55% mutation in sterlet, which might be due to different characteristics of embryos such as size, incubation temperature, cleavage pattern and ploidy level [53]. Interestingly, in sterlet when Cas9 protein instead of Cas9 mRNA was injected, mutation and survival rate of embryos increased by more than 90% [53]. Our present study was aimed to disrupt the *Ardnd1* to achieve sterility in sterlet (*Acipenser ruthenus*) to prepare a host for surrogate production in critically endangered sturgeon species. This study is consistent with Chen et al., [53], suggesting that Cas9 endonuclease can be efficiently applied in sturgeon having a higher ploidy level such as hexaploid or octoploid genomes.

Microinjection is an extensively used method of gene transfer in fish species due to its low cost, ease of visualization and high efficiency [54]. We have established this method in our lab to micro-manipulate sturgeon embryos. The sgRNA+Cas9 ribonucleoprotein complex was microinjected into 1 cell stage of sterlet eggs and this was repeated several times. Survival and hatching rates of embryos varied from batch to batch of eggs and also from different females; however, the mutation rate from phenotypic evidences was stable therefore proving the reliability of CRISPR/Cas9 in sterlet. Consistent with Chen et al., [53], low toxicity to embryos was found when different concentrations of sgRNAs and Cas9 endonuclease were injected. Some malformations in embryos in region where PGCs should originate were detected at 4 dpf in one batch of eggs from one female (Figure 1C); this is possibly because of double injection i.e., injection of sgRNA+Cas9 ribonucleoprotein complex into animal pole and FITC to label PGCs in the vegetal pole of embryos, and more likely due to off-target effects. The malformed embryos eventually died in the chorion membrane.

As expected, the number of PGCs was reduced in CRISPR/Cas9 treated embryos when compared with control embryos, however, in contrast, a few embryos with PGCs were still detectable in larvae until 22 dpf. Possible explanation can be mosaicism and/or physiological differences among embryos. Favoring the latter explanation, there might be differences in FITC labeling efficiency among embryos due to different chorion thickness, amount and/or depth/position germ plasm in embryo and also the depth/position of germ plasm in embryo [39]. Moreover, studies have showed that maternally supplied germ plasm and PGCs number vary among embryos within the same species [55,56], and the sturgeons are consistent with these findings [40,43].

We observed a healthy development of embryos after double injection until 22 dpf. Nevertheless, to guarantee the success of production of gametes via surrogacy, it is essential to check the development of sturgeons until the adult stage. It is important to confirm that complete gonads are formed in both of the sexes. In medaka (*Oryzias latipes*) and zebrafish (*Danio rerio*), complete removal of PGCs by using MO against *dnd1* led to production of all-male population in treated embryos [21,35]. However, in other fish species such as in loach (*Misgurnus anguillicaudatus*) and goldfish (*Carassius auratus*), sterilized embryos developed into both the sexes [22,23]. Linhartová and colleagues used AMO-*dnd1* in sturgeons to generate sterile host for surrogate production; however, sex determination after PGCs removal is still unclear [24]. Therefore, sex determination should not be biased in sturgeons when preparing the host for surrogate production. We have already established methods in our laboratory to prepare a germ cell free sterlet host. In our preset study, we compared three different methods to find out which method is more suitable in terms of embryo survival and hatching rates, and the removal of PGCs. Average lower survival and hatching rates were found as compared to CRISPR/Cas9 and UV240 irradiation when *Dnd1*-MO was injected as it is toxic to embryos [24].

Several previous studies in fish species have studied the functions of *dnd1* when both maternal as well as the zygotic mRNA are lost during early development. When CRISR/Cas9 was used against *dnd1* in Atlantic salmon, results showed that knockout (KO) fish were germ cell free in the F0 [33]. Thus, in the aforementioned studies, for the first time it has been revealed that CRISPR/Cas9 mediated knockouts of *dnd1* gene caused complete loss of the germ cells in F0 generation. Interestingly, maternal RNA was unable to compensate for loss of zygotic gene in F0 generation [33]. In sturgeons, the F0 mosaic embryos having maternal RNA compensating for zygotic gene loss could be targeted in the next generation to produce embryos having a complete loss of germ cells.

Host derived sperm or egg generation is not permitted in surrogate production technique to avoid the possibility of hybrids among offspring and consequent genetic contamination of target species. Due to complications in chromosomal pairing, interspecific hybrids of distantly related species are generally sterile. Nevertheless, hybrids of sturgeons from various species crosses tend to be viable and also fertile, even with different ploidy levels [57]. Therefore, 100% sterilization of the host is crucial in the surrogate production of sturgeons. We continue our work in this field to meet the promise that surrogate production in sturgeons is becoming feasible at a greater rate [24,39,43,58]. We trust that this cutting-edge technology, CRISPR/Cas9, will certainly be an invaluable tool for surrogate production of these IUCN red listed species.

## 5. Conclusions

In our present study, we have used CRISPR/Cas9 genome editing technology to achieve sterility in sterlet (*Acipenser ruthenus*) in order to prepare a host for surrogate production in sturgeons. The *dnd1* is essential for the formation and migration of PGCs in vertebrates, and its knock out in sterlet led to mismigration and/or absence of PGCs in the sterlet embryos, thus these sterile embryos can be potentially used as hosts for surrogacy in sturgeons.

Thereafter, we also compared three methods *viz.*, *Dnd1*-MO, UV irradiation and CRISPR/Cas9 that have been established in our lab to sterile sterlet. In terms of removal of PGCs, no significant difference was found among the compared methods; however, higher survival and hatching rates were found in CRISPR/Cas9, UV and MO, respectively.

## Figures and Tables

**Table 1:** Different concentrations of sgRNAs and Cas9 protein.

Treatment	Stage	No. of Embryos/Larvae	Min. No. of PGCs	Max. No. of PGCs	Mean	SD	*p* ***
Control	Neurula	29	12	86	44.14	21.04	0.006679
	22 dpf	14	12	41	26.57	10.30	
150/200 **	Neurula	30	0	20	7.23	5.85	0.00616
	22 dpf	20	0	12	5.90	4.78	
200/200 **	Neurula	21	0	19	6.90	6.94	0.9672
	22 dpf	19	0	12	3.68	4.06	
250/150 **	Neurula	21	0	21	7.23	6.70	0.6031
	22 dpf	18	0	15	5.22	4.85	

** sgRNA/Cas9 Concentrations in ng/µl; *** Wilcoxon test

**Table 2:** Comparison of CRISPR/Cas9, *Dnd1*-MO and UV240 methods.

Treatment	Stage	No. of Embryos/Larvae	Minimum No. of PGCs	Maximum No. of PGCs	Mean	SD	*p*
Control	Neurula	11	15	52	32.00	12.88	0.0004467
	22 dpf	8	51	92	71.88	16.78	
CRISPR/Cas9	Neurula	16	0	13	6.00	4.84	0.008104
	22 dpf	11	0	5	1.18	2.04	
Dnd1-MO	Neurula	20	0	16	3.30	5.59	0.7032
	22 dpf	9	0	12	5.56	4.77	
UV240	Neurula	20	0	16	6.05	5.96	0.3819
	22 dpf	19	0	18	4.89	6.13	

Wilcoxon test.

## Supplementary Materials

The following are available online at https://www.mdpi.com/2076-2615/9/4/174/s1, Figure S1: Generation of sgRNAs by overlap PCR.
