# Supplementary figures and images for: Dnd1 Knockout in Sturgeons By CRISPR/Cas9 Generates Germ Cell Free Host for Surrogate Production
Source: Animals (Basel). 2019 Apr 17;9(4):174. doi: 10.3390/ani9040174 (PMC6523263; doi:10.3390/ani9040174)

# Generation of gRNA by overlap PCR

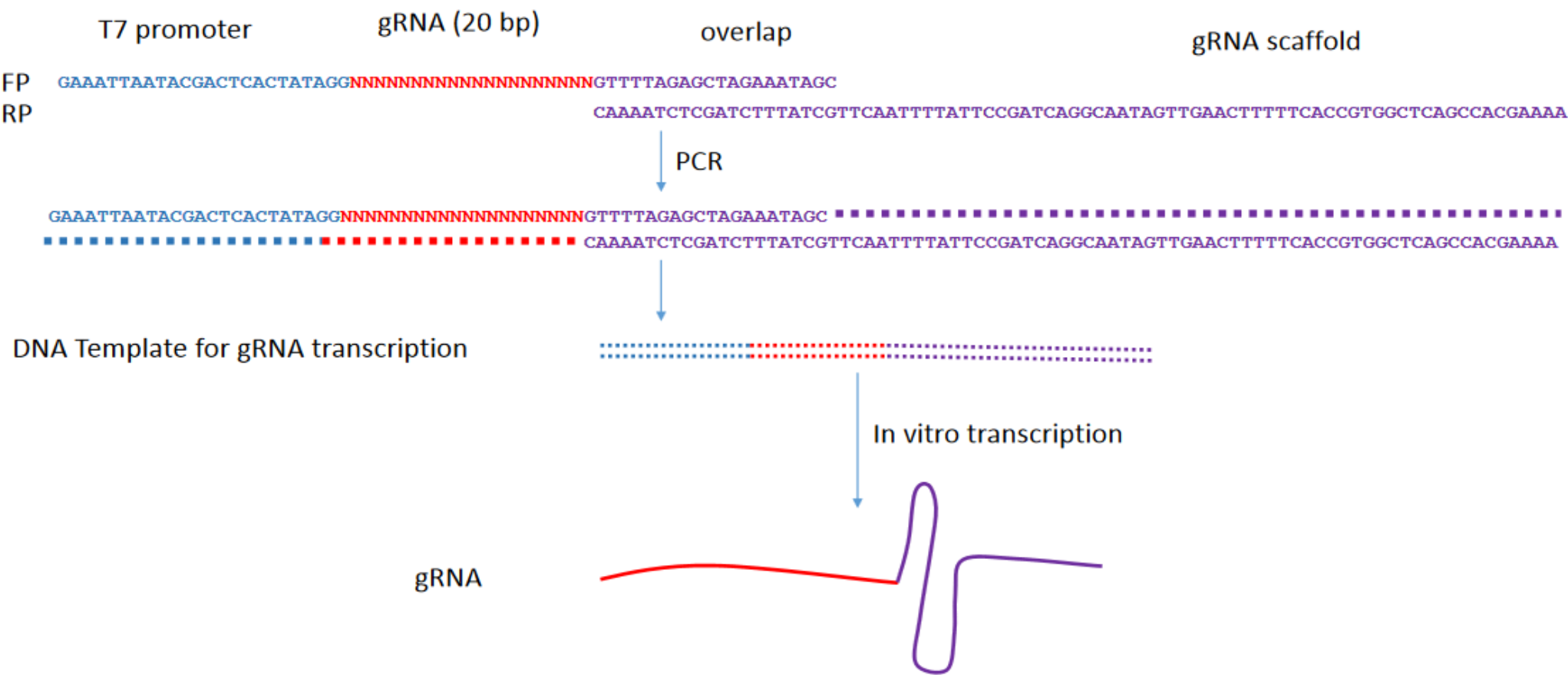

Supplement: Supplementary file 1 [file animals-09-00174-s001.pdf]
